# Prophylactic Laser and Cryotherapy in the Fellow Eye of Patients With Giant Retinal Tears: A Systematic Review and Meta-Analysis

**DOI:** 10.7759/cureus.99849

**Published:** 2025-12-22

**Authors:** Marisophie P Vasilakopoulou, Sofia Androudi, Ioannis Tsinopoulos, Panagiotis Stavrakas

**Affiliations:** 1 Ophthalmology, General Hospital of Chalkida, Chalkida, GRC; 2 Ocular Surgery, School of Medicine, Aristotle University of Thessaloniki, Thessaloniki, GRC; 3 Ophthalmology, University of Thessaly, Larissa, GRC; 4 Ophthalmology, School of Medicine, Aristotle University of Thessaloniki, Thessaloniki, GRC; 5 Ophthalmology, General Hospital of Athens "G. Gennimatas", Athens, GRC

**Keywords:** cryotherapy, fellow eye, giant retinal tear, laser prophylaxis, meta-analysis, retinal detachment, rhegmatogenous retinal detachment, systematic review

## Abstract

Giant retinal tears (GRTs) are associated with a substantial risk of rhegmatogenous retinal detachment (RD) in the fellow eye. Prophylactic retinal therapy, including laser photocoagulation and cryotherapy, is employed in particular individuals to reduce this risk; however, clinical consensus remains limited due to heterogeneous data. This systematic review and meta-analysis aimed to evaluate the effectiveness and safety of prophylactic treatment in preventing RD in the fellow eye of patients with unilateral GRT.

A comprehensive literature search was performed via PubMed, ScienceDirect, the Cochrane Library, and ClinicalTrials.gov through July 2025. Patients with unilateral GRT who underwent either observation or prophylactic treatment in their fellow eyes were the subjects of eligible studies. Extracted data included associated risk factors, ocular and systemic comorbidities that might predispose to GRT formation, demographics, treatment modality, follow-up duration, and the incidence of RD or new GRT formation. Risk of bias was assessed using the Joanna Briggs Institute checklist. A random-effects meta-analysis was performed to calculate the pooled relative risk for RD in treated versus untreated fellow eyes, while heterogeneity was evaluated with the I² statistic and publication bias with funnel plots and Egger's test.

A total of 803 fellow eyes were included across the eligible studies. Prophylactic treatment significantly reduced the risk of RD compared with observation (OR 0.14; 95% CI 0.07-0.28; p<0.001), with negligible heterogeneity (I²=0%). Subgroup analyses demonstrated consistent benefit for both laser-only and combined laser/cryotherapy approaches. Complications, when described, reduced the risk of RD during treatment, were typically mild, and included post-treatment retinal tears and epiretinal membrane formation; no adverse visual outcomes attributable to prophylactic treatment were consistently recorded.

Prophylactic laser photocoagulation alone or in combination with cryotherapy appears effective and safe in reducing RD risk in the fellow eyes of patients with GRT. These findings support the selective use of prophylactic treatment in high-risk eyes; however, high-quality prospective studies are required to validate the long-term advantages.

## Introduction and background

Giant retinal tears (GRTs) are extensive full-thickness retinal breaks encompassing over 90 degrees of the retinal circumference, commonly linked to posterior vitreous detachment and significant vitreoretinal tension [1,2]. GRTs are believed to develop from extensive vitreoretinal traction during posterior vitreous detachment, a process that elucidates their fast circumferential expansion and surgical complexity. Despite being relatively rare, constituting less than 1.5% of rhegmatogenous retinal detachments (RDs), GRTs signify a remarkably severe retinal condition due to their rapid development, complex surgical requirements, and poorer visual outcomes compared to standard rhegmatogenous RDs [3-7]. Clinically, GRTs are linked to sudden and extensive RD, frequently requiring complicated vitreoretinal surgery and posing a significant risk of irreversible loss of vision.

Although clinical care predominantly focuses on the affected eye, the involvement of the contralateral, initially asymptomatic eye is a significant concern. Bilateral GRTs have been observed in as many as 20% of individuals, with risk estimates differing based on follow-up time, underlying vitreoretinal conditions, and genetic predisposition [5,8,9]. Recent cohort studies indicate that the overall risk of rhegmatogenous RD in the contralateral eye may be significantly elevated, with reported rates approaching 30-35% throughout follow-up [5]. The risk is especially significant in patients with hereditary vitreoretinopathies, including Stickler syndrome, high myopia, or early disease onset, highlighting the medically relevant bilateral aspect of GRTs [8,10-12].

In consideration of the significant probability of fellow eye involvement, preventive intervention has been suggested as a possible approach for preventing subsequent RD. Prophylactic laser photocoagulation seeks to improve peripheral retinal adhesion and prevent the emergence of new retinal breaks or detachment [13]. Nonetheless, its function remains controversial. Certain surgeons support circumferential (360-degree) laser treatment for high-risk eyes, while others prefer observation due to apprehensions about overtreatment, peripheral vision field loss, and potential iatrogenic consequences [10,14-16]. Consequently, clinical practice exhibits significant variability, indicative of persistent doubt rather than agreement.

Although preventative measures are frequently addressed in therapeutic contexts, the data supporting them are insufficient and inconsistent. The majority of available data originate from retrospective cohort studies and limited case series, with no randomized controlled trials conducted so far to conclusively assess the efficacy of laser photocoagulation or cryotherapy in preventing fellow eye RD in patients with GRTs [4,11,17]. The diversity in study design, patient selection, outcome definitions, and follow-up duration complicates the interpretation of existing literature and undermines the reliability of current recommendations [18]. This ambiguity is particularly pertinent in patients with Stickler syndrome, who possess a significantly elevated lifetime risk of bilateral GRT and rhegmatogenous RD [19,20].

Consequently, an individual knowledge gap persists concerning the effectiveness of prophylactic therapy for the contralateral eye in significantly diminishing the risk of eventual RD or the emergence of a new GRT in patients with unilateral GRT. This systematic review and meta-analysis aims to synthesize current evidence regarding the incidence of fellow eye involvement and to evaluate the effectiveness of prophylactic interventions, including laser photocoagulation and cryotherapy, in preventing adverse retinal outcomes. This study aims to clarify this uncertainty to enhance evidence-based clinical decision-making for managing individuals at elevated risk for bilateral GRT.

## Review

Methods

Study Design and Protocol Registration

This systematic review and meta-analysis was conducted in accordance with the Preferred Reporting Items for Systematic Reviews and Meta-Analyses (PRISMA) 2020 guidelines. All procedures, including the search strategy, study selection, data extraction, risk of bias assessment, and statistical methodology, were defined a priori. **The review protocol was prospectively registered in the International Prospective Register of Systematic Reviews (PROSPERO) (ID: CRD420251241784).**

Search Strategy

The methodological approach for this literature review was based on the guidelines proposed by Paré et al. [18], which informed the design of the search strategy. We conducted a search of PubMed, ScienceDirect, the Cochrane Library, and ClinicalTrials.gov from their inception until June 30, 2025. The following search strategy was used in PubMed: (“giant retinal tear” OR GRT) AND (“fellow eye” OR bilateral) AND (prophylaxis OR “laser photocoagulation” OR cryotherapy). Similar search strategies, adapted to the syntax of each database, were applied in the remaining databases. Reference lists of all included studies were manually screened to identify additional relevant articles. Only full-text articles published in English were considered.

Data Extraction

Two reviewers separately extracted data utilizing a standardized form. Extracted variables covered study characteristics, fellow eye risk factors, type of prophylactic intervention, follow-up time, and verified clinical results. Conflicts were settled through consensus. The eligibility evaluation was conducted independently by two reviewers (M.V. and P.S.). Duplicates were detected and removed. The titles and abstracts were initially checked for inclusion, followed by a review and assessment of the entire submissions where required. Discrepancies were addressed and deliberated upon by consulting the corresponding author (M.V.).

Inclusion and Exclusion Criteria

Studies were eligible and qualified if they satisfied the following criteria: (1) observational study design, encompassing retrospective or prospective cohort studies and case series; (2) inclusion of patients diagnosed with unilateral GRT; (3) reporting of outcomes in the fellow eye, with or without prophylactic intervention; (4) provision of extractable data on at least one predefined outcome, including fellow eye rhegmatogenous RD, occurrence of GRT, visual acuity, or treatment-related complications; and (5) availability as full-text articles published in English. Studies were excluded based on the following criteria: (1) case reports, narrative reviews, editorials, letters, conference abstracts, or animal studies; (2) inclusion of fewer than five patients; (3) lack of separately reported fellow eye outcomes; or (4) inadequate data for extraction.

Outcomes

The outcomes of interest included rhegmatogenous RD due to GRTs, rhegmatogenous RD due to smaller, non-GRT-related retinal tears, and the final best-corrected visual acuity (BCVA). The occurrence of these outcomes in the contralateral eye was assessed as documented in the included studies.

Risk of Bias Assessment

The risk of bias was evaluated using the Joanna Briggs Institute (JBI) Critical Appraisal Checklists for Case Series and Cohort Studies. The evaluation focused on essential areas, such as patient selection, determination of exposures and outcomes, accuracy of follow-up, and reliability of reporting. The risk of bias evaluation was conducted separately by two reviewers, with discrepancies addressed through consensus. The outcomes of the risk of bias assessment were integrated into the qualitative synthesis, and the overall certainty of evidence was classified as low to moderate according to the retrospective design of the included studies.

Data Synthesis and Statistical Analysis

Statistical analysis was conducted in compliance with PRISMA guidelines. Data synthesis and statistical analysis were planned a priori. A quantitative meta-analysis was conducted for outcomes that provided homogeneous and extractable data across studies.

The meta-analysis included studies that provide comparative data on prophylactic therapies in contrast to observation. Effect estimates were represented as odds ratios (ORs) or relative risks (RRs) along with their respective 95% confidence intervals (CIs). Continuous outcomes, including ultimate BCVA, were summarized utilizing mean differences (MDs).

Statistical heterogeneity was assessed using Cochran's Q test and the I² statistic. Fixed- or random-effects models were utilized based on the level of heterogeneity. Subgroup analyses were conducted based on the prophylactic treatment method.

For outcomes where quantitative aggregation was impracticable due to clinical or methodological heterogeneity, inconsistent outcome definitions, or inadequate reporting of effect estimates, results were synthesized narratively in accordance with PRISMA recommendations and the Synthesis Without Meta-analysis (SWiM) guideline.

Publication bias was evaluated by funnel plot analysis and Egger's regression test, with careful interpretation owing to the restricted number of studies included. All analyses were bilateral, with statistical significance established at p<0.05. Statistical analyses were performed utilizing Review Manager (RevMan Version 5.4, The Cochrane Collaboration, London, England, United Kingdom), R software (meta package, R Foundation for Statistical Computing, Vienna, Austria), and Stata Version 19 (StataCorp LLC, College Station, Texas, United States).

Results

Study Selection

**Figure 1:**
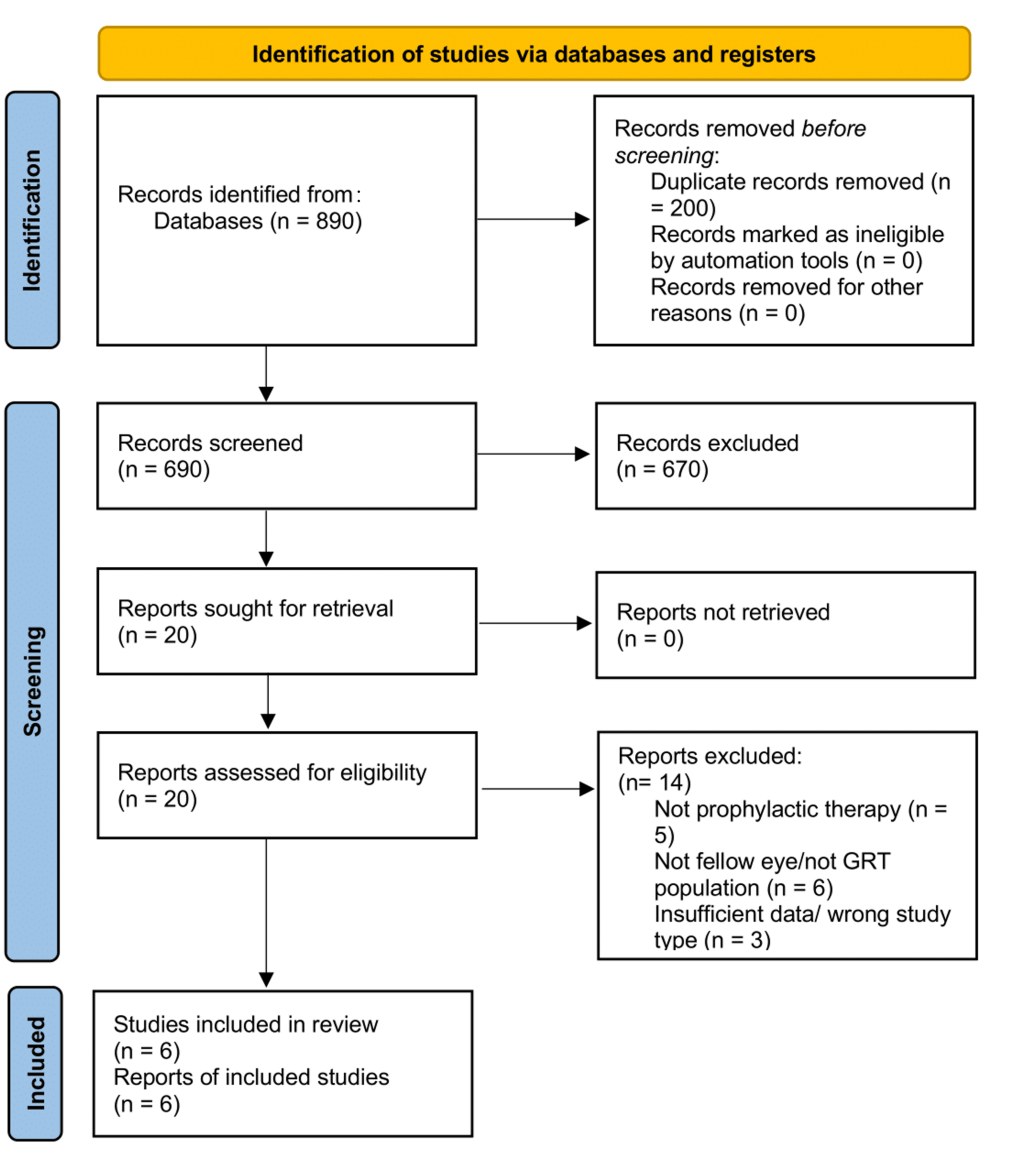
Article screening and retrieval following the PRISMA 2020 guidelines PRISMA: Preferred Reporting Items for Systematic Reviews and Meta-Analysis; GRT: giant retinal tear

The initial database search revealed a total of 890 documents (Figure 1). Following the removal of 200 duplicate data, 690 records remained and were evaluated based on titles and abstracts. Of these records, 670 were not included. Twenty reports were requested for full-text retrieval, all of which were successfully acquired and evaluated for eligibility. Fourteen reports were rejected for the following reasons: absence of prophylactic medication (n=5), lack of involvement of a colleague's eye in a GRT population (n=6), and inadequate data or flawed study design (n=3). In conclusion, six studies satisfied the inclusion criteria and were incorporated into the quantitative synthesis. 

Study Characteristics

Table 1 presents the characteristics of the included studies.

**Table 1:** Characteristics of the included studies RCCS: retrospective case-control study; PLT: prophylactic laser treatment; OBS: observational study; RCS (CS) or RCSrs: retrospective case series; GRT: giant retinal tear; NR: not reported; RCS: retrospective cohort study; EVBL: extended vitreous base laser; CS: case series NPL:

Author (Year)	Country	Study design	Fellow eyes (intervention/control)	Prophylactic intervention	Comparator	High-risk population	Follow-up (months)
Verhoekx et al., 2020 [14]	Netherlands	RCCS	78/51	360° laser photocoagulation	Observation	High myopia, lattice	110±44 (PLT); 103±43 (OBS)
Ripandelli et al., 2016 [15]	Italy	CS	98/62	360° laser photocoagulation	Historical controls	Young patients with GRT	37.2±16.3 (PLT); 43.5±19.8 (OBS)
Pitcher III et al., 2015 [21]	USA	CS	15/48	Focal laser to lesions	No prophylaxis	Lattice degeneration/breaks	NR
Al-Khairi et al., 2008 [22]	Saudi Arabia	CS	21/42	Laser or cryotherapy	Observation	Peripheral retinal pathology	29.7±26.7
Khanna et al., 2022 [10]	USA	RCS	129/101	360° laser (EVBL)	Observation/NPL	Stickler syndrome	Median 72 (up to 168)
Freeman, 1978 [8]	USA	OBS	65/171	Cryotherapy±scleral buckle	Untreated	High myopia, lattice	≥18 (up to 192)

Risk of Bias Summary

The methodological quality of the included studies was moderate. The majority of studies employed a retrospective approach, presenting potential concerns associated with selection bias and confounding by indication. The studies noted discrepancies in follow-up time and result reporting. No study was left out due to the risk of bias evaluation. A summary of the risk of bias assessment is presented in Table 2.

**Table 2:** Risk of bias assessment of the included studies Risk of bias was assessed using the Joanna Briggs Institute (JBI) Critical Appraisal Checklists for cohort studies and case series. OBS: observational study; CS: case series; RCS: retrospective cohort study; RCCS: retrospective case-control study.

Study (author, year)	Study design	Selection bias	Confounding	Outcome assessment	Follow-up adequacy	Reporting bias	Overall risk of bias
Freeman, 1978 [8]	OBS	Moderate	Moderate	Low	Moderate	Low	Moderate
Pitcher III et al., 2015 [21]	CS	Moderate	Moderate	Low	Low	Low	Moderate
Al-Khairi et al., 2008 [22]	CS	Moderate	Moderate	Moderate	Moderate	Low	Moderate
Ripandelli et al., 2016 [15]	CS	Moderate	Moderate	Low	Low	Low	Moderate
Verhoekx et al., 2020 [14]	RCCS	Low	Moderate	Low	Low	Low	Low-Moderate
Khanna et al., 2022 [10]	RCS	Low	Moderate	Low	Low	Low	Low-Moderate

Primary Outcome: RD Due to GRTs

Six studies, comprising a total of 803 patients, were included in the meta-analysis [8,10,14,15,21,22]. A quantitative meta-analysis was possible for this outcome due to comparable definitions and reporting across studies. The analysis demonstrated that primary prophylactic treatment (prophylactic laser treatment (PLT), PLT/cryotherapy (Cryo), or Cryo alone) was associated with a significantly lower likelihood of developing rhegmatogenous RD in the fellow eye, while new GRT occurrence was reported descriptively when available in the fellow eye compared with control groups.

The pooled OR for RD occurrence was 0.14 (95% CI: 0.07-0.28), indicating a markedly reduced risk in the intervention group (p<0.001). No heterogeneity was detected among the included studies (I²=0%), supporting the robustness of the fixed-effects model.

The largest weight in the analysis was attributed to the study by Khanna et al. (35.72%) [10], whereas the studies by Pitcher III et al. [21] and Al-Khairi et al. contributed less due to smaller sample sizes [22].

**Figure 2:**
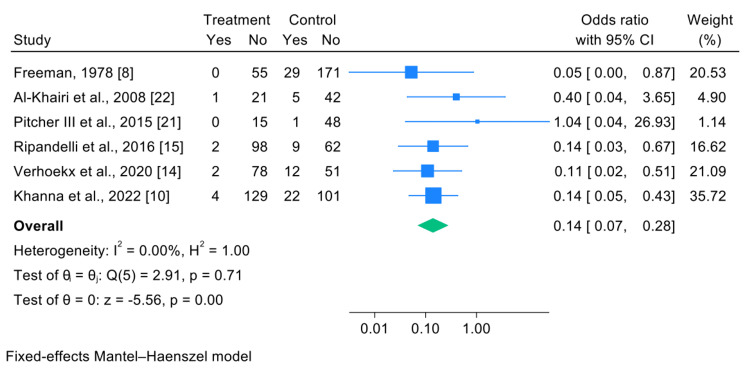
Forest plot comparing the probability of developing RD due to GRTs between the intervention and control groups RD: retinal detachment; GRTs: giant retinal tears

The forest plot (Figure 2) illustrates that all studies, except one, reported OR values below 1, indicating a consistent beneficial effect of the intervention. The study by Pitcher III et al. was the only one that found an OR greater than 1 [21], but this result was not statistically significant because the confidence interval was so wide (95% CI: 0.04-26.93).

Overall, the quantitative synthesis indicates a significant reduction in the incidence of fellow eye RD secondary to GRT following prophylactic intervention.

Subgroup Analysis by Prophylactic Modality 

Subgroup analysis revealed that both PLT alone and combined PLT/Cryo (or Cryo alone) were similarly effective in preventing GRT-related RD. For PLT alone, the pooled OR was 0.12 (95% CI: 0.04-0.31; p<0.001; I²=0%) based on four studies [8,21,15,14]. For PLT/Cryo or Cryo alone, the pooled OR was 0.17 (95% CI: 0.07-0.46; p<0.001; I²=0%) from two studies [22,10]. The between-subgroup difference was not statistically significant (p for subgroup difference=0.57), indicating that both approaches achieve comparable prophylactic benefit when appropriately applied. This finding is notable, as earlier reports had suggested possible differences in efficacy or complication rates between modalities.

Publication Bias

**Figure 3:**
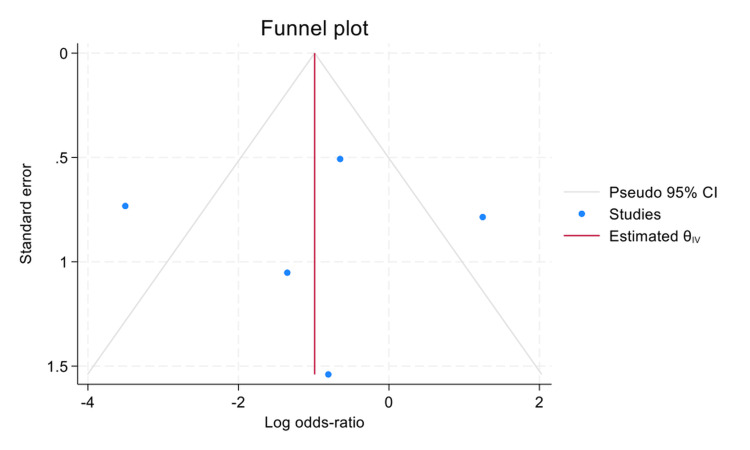
Funnel plot assessing publication bias for RD due to GRT RD: retinal detachment; GRT: giant retinal tear

With regard to publication bias (Figure 3), visual inspection of the funnel plot revealed relative symmetry of studies around the pooled effect estimate (vertical line), without evidence of marked asymmetry, suggesting no strong indication of publication bias. This was further supported by Egger's regression test, which showed no statistically significant small-study effects (β₁=0.79; SE=1.17; z=0.68; p=0.498). The non-significant p-value (>0.05) reinforces the likelihood of minimal publication bias. However, the small number of included studies (n=6) limits the statistical power of these assessments, and caution is advised in interpretation.

Stratified Analysis

**Figure 4:**
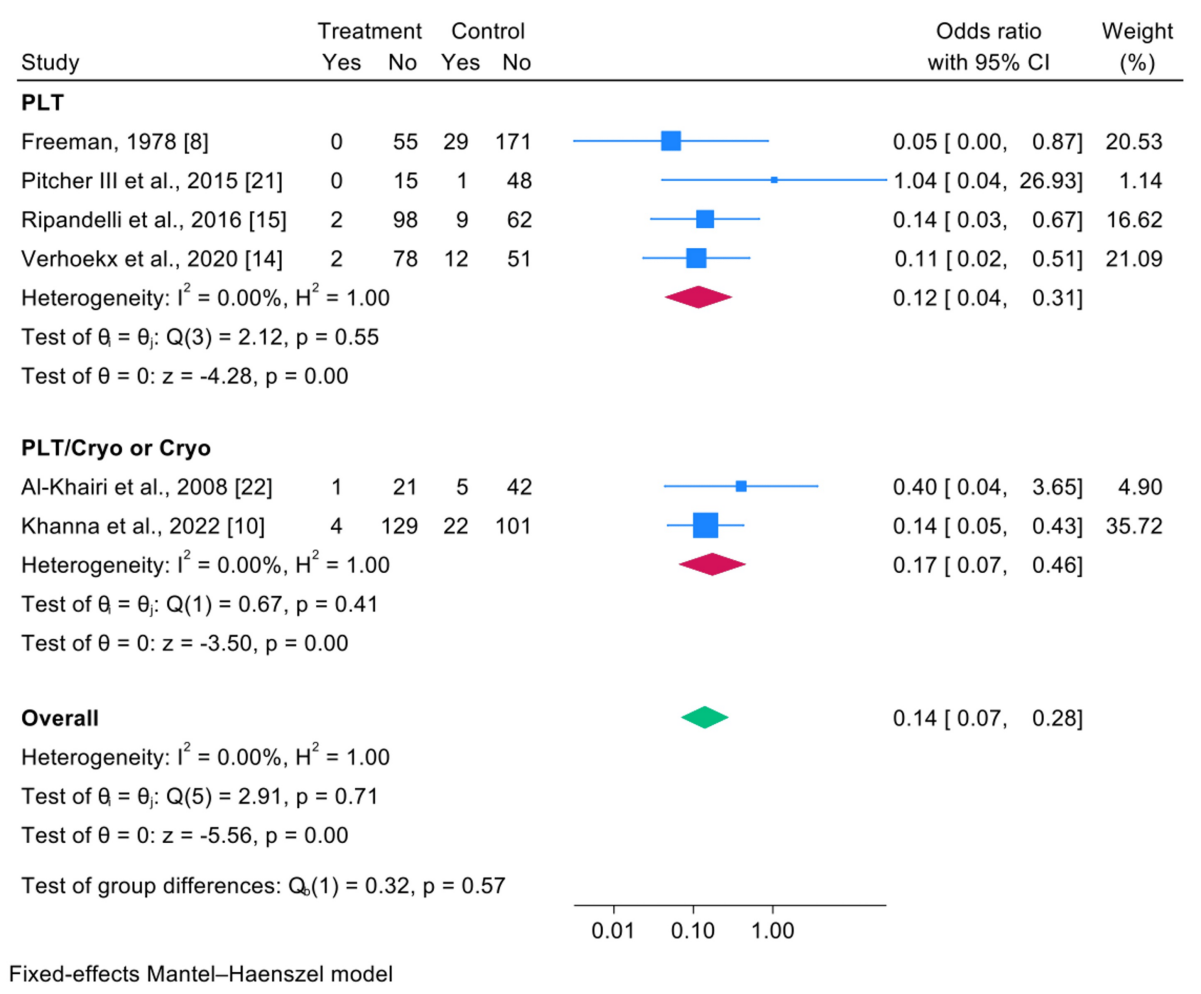
Stratified forest plot comparing treatment groups regarding the probability of developing RD due to GRTs RD: retinal detachment; GRTs: giant retinal tears

The studies were subsequently divided according to the treatment approach to identify any differences. The results are presented in Figure 4.

For PLT (laser treatment), four studies were included [8,21,15,14]. The pooled OR was 0.12 (95% CI: 0.04-0.31; p<0.001). No significant heterogeneity was observed (I²=0%; Q(3)=2.12; p=0.55).

For studies using PLT/Cryo or Cryo alone, two were included [22,10]. The pooled OR was 0.17 (95% CI: 0.07-0.46; p<0.001). Again, no within-group heterogeneity was detected (I²=0%; Q(1)=0.67; p=0.41).

The between-subgroup difference was not statistically significant (Q(1)=0.32; p=0.57), indicating that effectiveness did not differ substantially between the two treatment approaches (PLT vs. PLT/Cryo or Cryo alone). Both treatment modalities demonstrated similarly significant positive effects.

Secondary Outcome: RD Due to Smaller Retinal Tear

**Figure 5:**
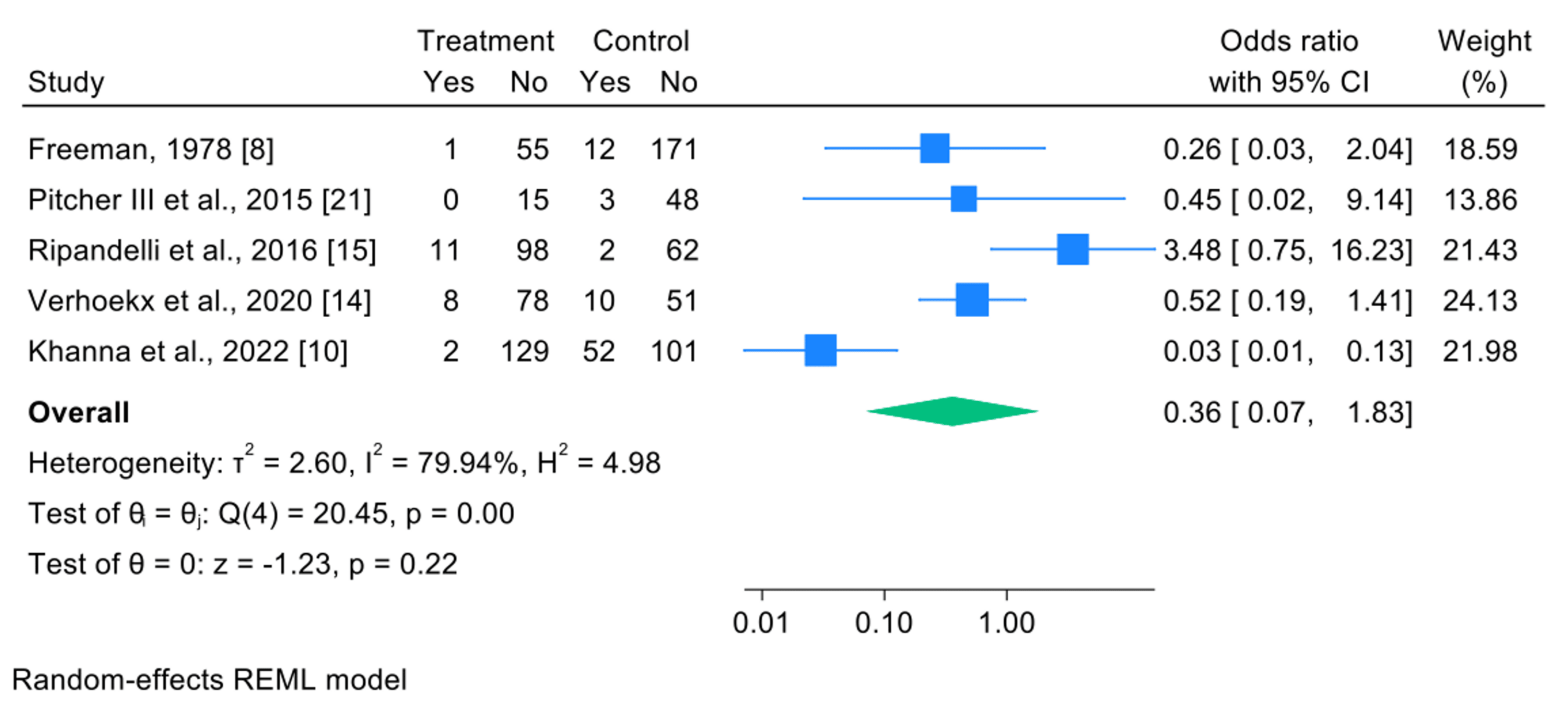
Forest plot comparing the risk of RD due to smaller retinal breaks between the treatment groups RD: retinal detachment

The meta-analysis included five studies reporting cases of RD due to smaller retinal tears (non-GRT-related tears) [8,10,14,15,21]. The pooled estimate was as follows: OR=0.36, 95% CI: 0.07-1.83, and p=0.22 (not statistically significant). Although the OR suggests a favorable trend in favor of the intervention (reduced likelihood of RD), the result was not statistically significant. The wide confidence interval indicates uncertainty in the estimate (Figure 5).

Heterogeneity across studies was high (I²=79.94%; Q(4)=20.45; p<0.001). This significant heterogeneity indicates that variations in study results are not merely attributable to random fluctuations. Potential sources of heterogeneity may include differences in surgical technique (PLT vs. Cryo), varying criteria for documenting RD, and heterogeneity in the anatomical location of the retinal tears. The specific characteristics of non-GRT-related retinal tears, such as the number of tears, clock-hour extent, and correlation with lattice degeneration, were inconsistently documented in the papers reviewed, precluding quantitative evaluation.

The study by Ripandelli et al. [15] was the only one with an OR of >1 (3.48; 95% CI: 0.75-16.23) [15], while the remaining studies yielded values <1.

**Figure 6:**
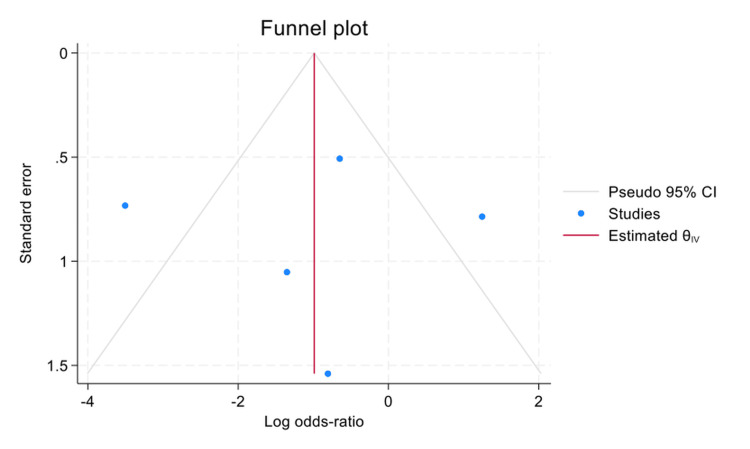
Funnel plot assessing publication bias for RD due to smaller retinal tear RD: retinal detachment

The funnel plot (Figure 6) shows a relatively symmetrical distribution of study points around the central estimate (θ), without evident asymmetry, indicating no strong likelihood of small-study effects. Egger's test confirmed this: β₁=-0.26, SE=1.33, z=-0.20, and p=0.8437. The very high p-value (>0.05) provides no statistically significant evidence of publication bias. However, as with previous analyses, the small number of studies (n=5) limits the statistical power and warrants caution in interpreting the results.

Secondary Outcome: Visual Acuity (BCVA)

**Figure 7:**
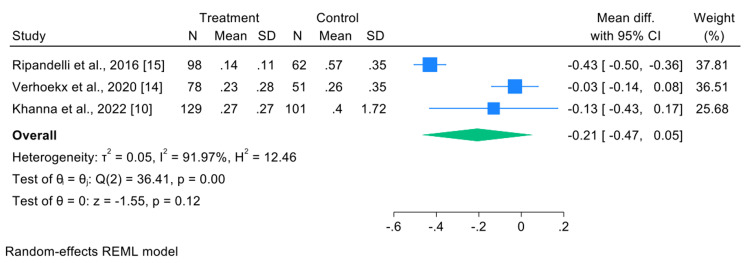
Forest plot comparing visual acuity outcomes between the intervention and control groups

The analysis included three studies that reported mean final BCVA [10,14,15]. The pooled result showed a mean difference of −0.21 (95% CI: −0.47 to 0.05; p=0.12) (Figure 7). Although the difference favored the intervention group (lower BCVA values indicate better visual acuity in many scales), the result was not statistically significant.

Very high heterogeneity was present (I²=91.97%; Q(2)=36.41; p<0.001), indicating that over 90% of the observed variability was due to true differences between studies rather than random error. Possible sources of heterogeneity include differences in BCVA measurement timepoints, variations in calculation methods, heterogeneity in patient populations, and differences in treatment techniques.

The study by Ripandelli et al. [15] showed a statistically significant difference in favor of the intervention (mean difference=−0.43; 95% CI: −0.50 to −0.36), whereas the other two studies did not demonstrate significant differences.

Fellow Eye Outcomes

Across the included studies, prophylactic treatment of the fellow eye demonstrated a consistent trend toward decreased incidence of rhegmatogenous RD and new GRT formation. The effect size differed based on the treatment type, underlying pathology, and follow-up duration. Laser-based prophylaxis often had the most significant protective effect, especially in high-risk populations, including individuals with Stickler syndrome [19,20]. Visual acuity outcomes were less consistently reported and showed substantial variability, indicative of disparities in measuring scales and assessment time.

Narrative Synthesis of Outcomes

For outcomes inappropriate for quantitative aggregation, results were synthesized narratively in accordance with PRISMA and SWiM guidelines. Pooling proved unfeasible owing to substantial clinical and methodological heterogeneity, discrepancies in outcome definitions and follow-up, and inadequate reporting of standardized effect measurements.

Evidence Summary

In the studies analyzed, prophylactic therapy of the contralateral eye was consistently linked to a reduced occurrence of rhegmatogenous RD resulting from GRTs. Conversely, the results regarding RD from smaller non-GRT-related tears and final BCVA were less consistent, affected by variability in research populations, therapies, and reporting, leading to increased uncertainty for these secondary outcomes.

Strengths and Limitations

This review systematically summarizes information from clinically pertinent studies assessing prophylactic therapy of the contralateral eye in patients with unilateral GRTs, incorporating both historical and current data, including both cohort and multicenter studies. Quantitative synthesis was conducted when possible, facilitating an objective comparison of preventive techniques, including circumferential laser photocoagulation and cryotherapy. The present study is the first systematic review to evaluate fellow eye outcomes across various prophylactic strategies in this distinctly high-risk demographic.

The majority of the included studies were retrospective, exposing inherent risks of selection bias, inconsistent follow-up, and insufficient reporting. Significant clinical variability was noted among studies regarding patient characteristics, underlying etiologies (e.g., Stickler syndrome, high myopia), prophylactic methods, extent of laser therapy, follow-up time, and definitions of outcomes, especially for visual outcomes. Numerous studies, including broader or non-GRT-specific populations, were incorporated mainly for contextual reasons, thus decreasing the accuracy of risk-benefit extrapolation. Recent literature consistently presents incongruous and primarily observational findings about contralateral eye prophylaxis, with persistent ambiguity in patient selection despite advancements in surgical procedures [23]. The limited number of qualified studies reduced statistical power for secondary outcomes, including visual acuity and non-GRT-related retinal detachment. Authoritative studies, such as those by the Cochrane Collaboration [11], emphasize that the lack of randomized controlled trials, primarily due to the rarity and complexity of GRT, constrains the overall certainty of the existing data. Limiting to English-language articles may create linguistic bias, and recent scoping studies illustrate significant diversity in reported risk factors, treatment methods, and results in GRT-related retinal detachment [24].

Discussion

This systematic review and meta-analysis synthesizes the available evidence for prophylactic treatment of the fellow eye in patients with unilateral GRTs. The pooled analysis of six studies (n=803 eyes) showed that prophylactic laser photocoagulation (PLT), PLT combined with cryotherapy, or cryotherapy alone was associated with a markedly reduced risk of RD due to GRT compared with observation or non-interventional management. The overall OR was 0.14 (95% CI: 0.07-0.28; p<0.001; I²=0%) (Figure 1). This indicates an 86% relative risk reduction, with no detected heterogeneity across studies, supporting the robustness of this effect.

It is important to note that many of the previous studies featured in this review, especially small older case series and non-GRT-specific publications, were performed in a prior surgical era and may not accurately represent modern vitreoretinal methods or current patient selection criteria. Consequently, these studies are understood mostly within a historical framework, offering essential insights into preventive principles rather than conclusive direction for contemporary therapeutic treatment.

For RD due to smaller, non-GRT-related retinal tears, a pooled analysis of five studies yielded an OR of 0.36 (95% CI: 0.07-1.83; p=0.22) (Figure 4). Although the point estimate favored prophylaxis, the result was not statistically significant, and heterogeneity was high (I²=79.94%; p<0.001). The study by Ripandelli et al. was the only one with an OR of >1 (3.48; 95% CI: 0.75-16.23), possibly due to a broader inclusion of lattice degeneration and non-standardized prophylactic coverage. This variability suggests that prophylaxis may be less uniformly effective for preventing smaller secondary tears, potentially reflecting differences in pathogenesis compared to primary GRT formation.

Visual outcomes, measured as final BCVA, were reported in three studies. The pooled mean difference was -0.21 (95% CI: -0.47 to 0.05; p=0.12) (Figure 6), favoring the prophylaxis group but without statistical significance. Heterogeneity was very high (I²=91.97%; p<0.001), indicating substantial methodological and population differences. Ripandelli et al. reported a statistically significant BCVA benefit (mean difference=-0.43; p<0.001), which may reflect earlier intervention in the disease course or more rigorous follow-up protocols in their cohort.

Publication bias assessment for the GRT-related RD outcome (Figure 2) showed symmetrical funnel plot distribution, and Egger's test was non-significant (p=0.498), suggesting no strong evidence of small-study effects. Similar findings were observed for the smaller-tear RD outcome (Figure 5; Egger's p=0.8437). However, the limited number of included studies (n=6-5 per outcome) restricts the power of these tests, and results should be interpreted with caution.

These results have significant clinical implications. The prophylactic advantage reported is unlikely to be consistent among all patients and seems to fluctuate based on baseline risk. In high-risk individuals, including those with hereditary vitreoretinopathies [25,26], high myopia, lattice degeneration, or a younger age at initial GRT, the number needed to treat (NNT) to avert a second eye detachment is likely minimal, thereby endorsing the evaluation of prophylactic intervention. In lower-risk individuals, the possibility of visual field loss or iatrogenic effects from extensive prophylactic treatment must be carefully considered in relation to the absolute risk reduction, highlighting the necessity for personalized decision-making.

## Conclusions

Prophylactic intervention in the fellow eye of patients with unilateral GRTs significantly diminishes the likelihood of eventual RD, no matter the use of laser photocoagulation alone or in combination with cryotherapy. Nonetheless, sustained enhancement in secondary outcomes, such as RD from small tears or final visual acuity, was not observed. The interpretation of these findings demands caution, as the evidence primarily stems from retrospective observational studies characterized by variability in baseline risk, treatment methods, and follow-up duration, leading to low overall certainty. Prophylactic treatment may be especially pertinent for some high-risk populations, such as individuals with inherited vitreoretinopathies (e.g., Stickler syndrome). Thorough prospective studies are essential for clarifying long-term functional results to improve risk-based patient selection.
